# Supplementary material for: Multivariate and Geometric Morphometrics Reveal Morphological Variation Among Sinibotia Fish
Source: Biology (Basel). 2025 Sep 2;14(9):1177. doi: 10.3390/biology14091177 (PMC12467238; doi:10.3390/biology14091177)
Supplement: Supplementary file 1 [file biology-14-01177-s001.zip › biology-3815941-SI.pdf]

Table S1. ANOVA table for multivariate morphological characters among *Sinibotia* fishes

Variable	*F*	*p*	Variable	*F*	*p*	Variable	*F*	*p*
HL	40.642	0.000	CFL	129.413	0.000	D_{4-5}	32.777	0.000
ED	48.389	0.000	TL	121.306	0.000	D_{4-6}	3.032	0.020
SnL	20.456	0.000	D_{1-2}	28.191	0.000	D_{4-7}	124.305	0.000
HBE	30.009	0.000	D_{1-10}	20.844	0.000	D_{4-8}	144.148	0.000
HD	135.721	0.000	D_{2-3}	52.686	0.000	D_{4-9}	194.196	0.000
NSD	13.563	0.000	D_{2-8}	15.873	0.000	D_{4-10}	43.978	0.000
BD	252.310	0.000	D_{2-9}	42.449	0.000	D_{5-6}	142.643	0.000
DFL	48.469	0.000	D_{2-10}	81.037	0.000	D_{5-7}	61.342	0.000
PFL	99.873	0.000	D_{3-4}	216.707	0.000	D_{5-8}	16.610	0.000
VFL	63.324	0.000	D_{3-7}	239.706	0.000	D_{6-7}	61.342	0.000
AFL	62.308	0.000	D_{3-8}	171.279	0.000	D_{7-8}	36.370	0.000
CPL	17.811	0.000	D_{3-9}	227.354	0.000	D_{8-9}	21.424	0.000
CPH	84.549	0.000	D_{3-10}	37.272	0.000	D_{9-10}	28.21	0.000

Table S2. Results of discriminant function analysis based on multivariate morphological characters

Methods	Species	Predicted group membership					Discriminant accuracy/%	Integrated discriminant accuracy//%
		S. superciliaris	*S. reevesae*	*S. robusta*	*S. pulchra*	*S. zebra*		
Stepwise discriminant	*S. superciliaris*	30	0	0	0	0	100	100
	S. reevesae	0	30	0	0	0	100	
	S. robusta	0	0	32	0	0	100	
	S. pulchra	0	0	0	30	0	100	
	S. zebra	0	0	0	0	28	100	
Cross-validation	*S. superciliaris*	30	0	0	0	0	100	100
	S. reevesae	0	30	0	0	0	100	
	S. robusta	0	0	32	0	0	100	
	S. pulchra	0	0	0	30	0	100	
	S. zebra	0	0	0	0	28	100	

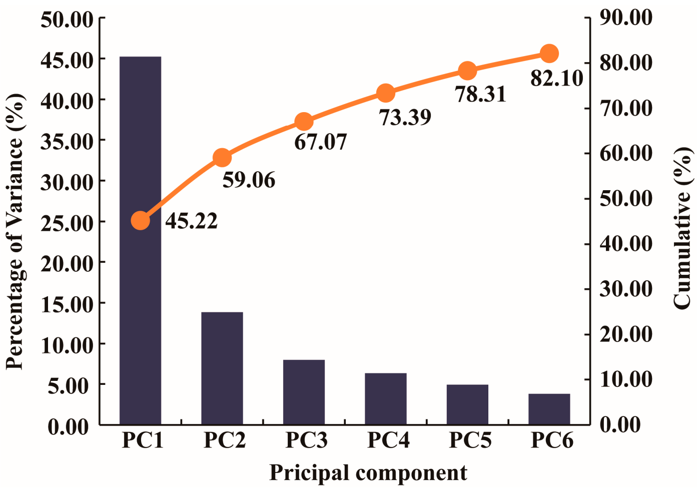

Figure S1. Scree plot of the PCA based on multivariate morphological characters.

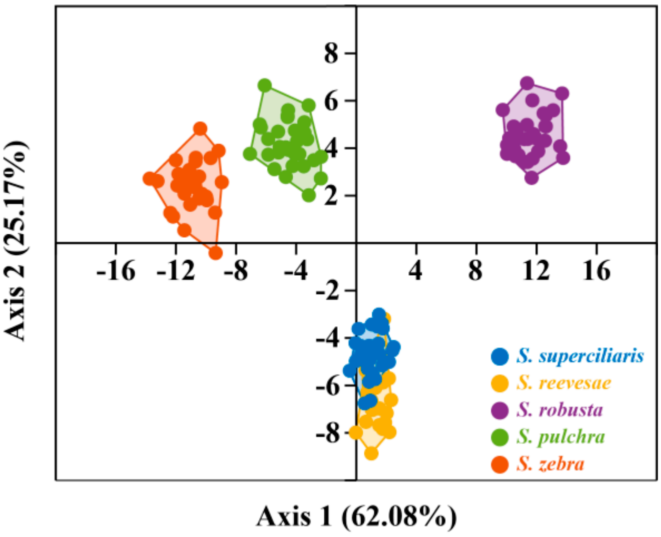

Figure S2. DFA plot for the five *Sinibotia* species base on 34 landmarks of the lateral view.

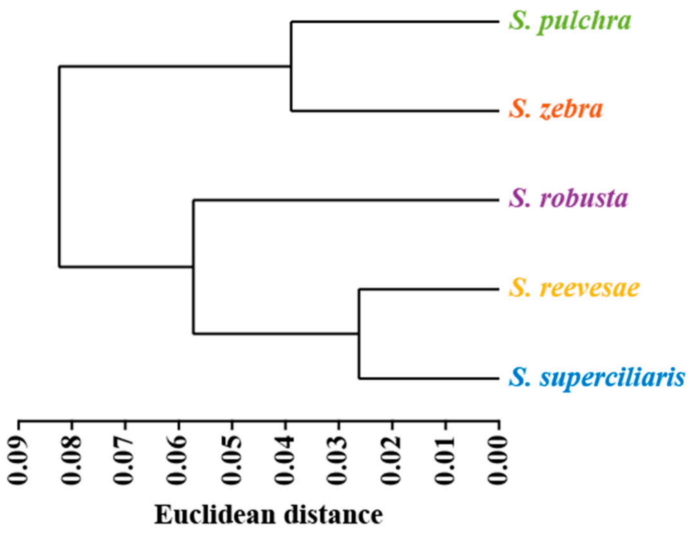

Figure S3. Dendrogram derived based on 34 landmarks of the lateral view.
